# Optical Properties of Magnesium-Zinc Oxide for Thin Film Photovoltaics

**DOI:** 10.3390/ma14195649

**Published:** 2021-09-28

**Authors:** Mohammed A. Razooqi Alaani, Prakash Koirala, Adam B. Phillips, Geethika K. Liyanage, Rasha A. Awni, Dhurba R. Sapkota, Balaji Ramanujam, Michael J. Heben, Stephen K. O’Leary, Nikolas J. Podraza, Robert W. Collins

**Affiliations:** 1 Wright Center for Photovoltaics Innovation & Commercialization, Department of Physics & Astronomy, University of Toledo, Toledo, OH 43606, USA; prakash.koirala@utoledo.edu (P.K.); adam.phillips@utoledo.edu (A.B.P.); Geethika.Liyanage@rockets.utoledo.edu (G.K.L.); rasha.awni@utoledo.edu (R.A.A.); dhurbaraj.sapkota@rockets.utoledo.edu (D.R.S.); balaji.ramanujam@utoledo.edu (B.R.); michael.heben@utoledo.edu (M.J.H.); 2 School of Engineering, The University of British Columbia Okanagan, 3333 University Way, Kelowna, BC V1V 1V7, Canada; stephen.oleary@ubc.ca

**Keywords:** (Mg,Zn)O, optical properties, spectroscopic ellipsometry, CdTe photovoltaics

## Abstract

Motivated by their utility in CdTe-based thin film photovoltaics (PV) devices, an investigation of thin films of the magnesium-zinc oxide (Mg*_x_*Zn_1−*x*_O or MZO) alloy system was undertaken applying spectroscopic ellipsometry (SE). Dominant wurtzite phase MZO thin films with Mg contents in the range 0 ≤ *x* ≤ 0.42 were deposited on room temperature soda lime glass (SLG) substrates by magnetron co-sputtering of MgO and ZnO targets followed by annealing. The complex dielectric functions ε of these films were determined and parameterized over the photon energy range from 0.73 to 6.5 eV using an analytical model consisting of two critical point (CP) oscillators. The CP parameters in this model are expressed as polynomial functions of the best fitting lowest CP energy or bandgap *E*_0_ = *E_g_*, which in turn is a quadratic function of *x*. As functions of *x*, both the lowest energy CP broadening and the Urbach parameter show minima for *x* ~ 0.3, which corresponds to a bandgap of 3.65 eV. As a result, it is concluded that for this composition and bandgap, the MZO exhibits either a minimum concentration of defects in the bulk of the crystallites or a maximum in the grain size, an observation consistent with measured X-ray diffraction line broadenings. The parametric expression for ε developed here is expected to be useful in future mapping and through-the-glass SE analyses of partial and complete PV device structures incorporating MZO.

## 1. Introduction

Cadmium telluride (CdTe) thin film solar cell performance has advanced remarkably over the past decade with significant increases in both small area device and large area module efficiencies [1]. One focus of the reported improvements has been to increase photogenerated carrier collection through the incorporation of composite or alloyed window and graded absorber layers at the front of the solar cell. Successful approaches include a thinner cadmium sulfide (CdS) or cadmium sulfide-oxide (CdS:O) window layers made possible by a photoelectronically active CdTe_1__−*x*_Se*_x_* graded alloy serving as the absorber layer component. With such structures at the front of the solar cell, significant improvements were achieved in the short circuit current density (*J*_SC_) of the device but with little if any improvement in its open circuit voltage (*V*_OC_) [2,3,4,5,6,7,8]. Over these last several years, it has been reported that resistive oxide materials can improve the open circuit voltage of the CdTe solar cell by reducing interfacial recombination at the semiconductor *n*-*p* junction [9,10,11]. In fact, it has been demonstrated that a high-quality junction can be achieved between a high resistivity transparent (HRT) layer and a *p*-type CdTe absorber layer even without the thin CdS or CdS:O layer, depending on the precise band alignment [12] and the front barrier height [13] at the HRT/(absorber layer) interface.

This recent research has shown that ensuring proper energy band alignment at the front of the CdTe solar cell is a critical approach for enhancing *V*_OC_ of the device. Mg*_x_*Zn_1−*x*_O (MZO) has attracted greater attention than other potential HRT layers in such applications due to the flexibility it provides for engineering the interface between the HRT and solar cell layers by tuning the bandgap (*E_g_*) of the MZO through adjustment of the Mg content (*x*) [10,11,12,13,14,15]. In fact, synthesizing a solid solution of ZnO (*E_g_* = 3.3 eV) with wider bandgap MgO (*E_g_* = 7.8 eV) is a proven method for engineering wide bandgap semiconductor layers for a variety of applications [16,17,18,19]. Thus, in the case of thin film CdTe solar cells, applying MZO in an HRT bi-layer of SnO_2_/MZO results in interfaces that act to facilitate electron extraction by minimizing interface recombination [12]. Finally, it is found that the underlying MZO has a larger grain size in contact with the overlying CdTe than does the traditional CdS window layer, thus improving the absorber layer junction morphology when used in direct contact with CdTe [20].

It was observed that when a MZO thin film is applied in a heterojunction with a *p*-type semiconductor, the conduction band minimum of the MZO shifts closer to the vacuum level with increasing Mg content *x* [19,21]. This shift can increase *V*_OC_ in a heterojunction solar cell by reducing the conduction band offset [13,19,22,23]. In general, however, MZO thin films may exhibit poor stability due to the possible presence of multiple phases including metastable crystal structures [22]. Although the stable phases of ZnO and MgO are wurtzite and cubic rocksalt, respectively, metastable phases of each compound may occur in the alloy especially when the layers are deposited in energetic physical vapor deposition processes. This makes it more difficult to obtain the desired homogeneous single phase with the desired stoichiometric ratio, a well-defined and tunable bandgap, and a sharp Urbach tail [22]. Consequently, many deposition processes and variables have been explored in previous research to optimize the quality of MZO thin films as well as their resulting performance in devices. Magnetron sputtering facilitates optimization of the MZO film composition and structure through control of the target material composition, as well as the species flux, surface mobility, and level of ion bombardment via adjustment of the applied power, substrate temperature, and sputtering gas pressure settings, respectively [17,24,25]. Post-deposition treatments, such as surface passivation, annealing, and light soaking, serve as further approaches to enhance the crystallinity and decrease the defect concentration in MZO thin films [26,27,28].

To date, the effect of varying the Mg content of MZO films on solar cell device performance has yet to be elucidated in detail. Further investigations are required to identify and understand correlations between the MZO film optical properties and the performance of the CdTe devices that incorporate MZO as either an HRT or a window layer. In this study, a series of complementary measurements were performed to facilitate such correlations. Toward this goal, we have determined the composition, as well as the structural and optical properties of MZO layers, focusing on the thin film CdTe photovoltaics (PV) applications of these layers. The MZO films were deposited by radio-frequency (RF) magnetron co-sputtering from MgO and ZnO targets. Increasing the power applied to the MgO target relative to that applied to the ZnO target enables the replacement of an atomic fraction *x* of Zn by Mg in ZnO to form the Mg*_x_*Zn_1−*x*_O alloy. Prior to the measurement of their properties, MZO films deposited at room temperature on soda lime glass (SLG) substrates were subjected to a thermal cycle that such films would experience as an HRT layer in CdTe solar cell fabrication. This approach results in a data set of basic properties of relevance for the CdTe PV applications of greatest interest.

The thermally cycled SLG/MZO samples studied here span the range of Mg content *x* from *x* = 0 to 0.42, as measured by energy dispersive X-ray spectroscopy (EDS) analysis. Over this range, the stable phase of ZnO, i.e., the wurtzite phase, continues to be the dominant phase of the alloys, as determined from their X-ray diffraction (XRD) patterns. In order to investigate the effect of Mg incorporation on the optical properties and energy bandgap, these samples were measured by spectroscopic ellipsometry (SE), providing complete optical characterization from the near-infrared to the ultraviolet (NIR-UV-SE) spanning the photon energy range from 0.73 to 6.5 eV. The SE spectra were analyzed by least-squares regression using structural and optical models with photon energy independent variables of thicknesses that describe the structure and oscillator parameters that describe the complex dielectric function spectra. The structural model includes SLG/MZO interface, MZO bulk, and MZO surface roughness layers and the optical model includes two critical point oscillators, one defining the MZO bandgap and the other located above the measured spectral range. Although several of the oscillator parameters were fixed as a function of the Mg content *x*, the best fitting variable oscillator parameters of the deposition series were smooth functions of the composition. From these best fitting analyses, an increase in bandgap energy from *E_g_* = 3.26 to 3.83 eV was observed for the wurtzite MZO over the range from *x* = 0 to 0.42. In addition, the bandgap CP broadening and the Urbach parameter showed minima for *x* ~ 0.3, which corresponded to a bandgap of 3.65 eV, suggesting either a minimum in the defect concentration in the crystallite bulk or a maximum in the grain size for this composition. In fact, all best fitting variable CP parameters when plotted versus the best fitting bandgap, which is more accurately determined than *x*, exhibit systematic behavior that can in turn be fit with polynomial functions. Thus, the polynomial coefficients serve as a database that enable prediction of the MZO complex dielectric function for any specified bandgap. Such a database is expected to have important future applications in the metrology of PV device structures incorporating MZO.

This article is organized in the following manner. Section 2 provides the experimental details of materials fabrication along with the instrumentation used for NIR-UV-SE. Section 3 is divided into three sub-sections each presenting experimental and modeling results as well as accompanying discussion. Section 3.1 and Section 3.2 present the results of structural and compositional analyses of the annealed SLG/MZO substrate/films by XRD and EDS, respectively. Section 3.3 is the key foundational component of the research and presents a parametric description of the complex dielectric function of the MZO films as a function of bandgap. The results of this section enable various metrological applications that will be presented in future reports. Applications of these results, and further means of characterizing our samples, are then discussed in Section 3.4. Finally, Section 4 provides a summary of the major conclusions of this work.

## 2. Materials and Methods

MZO thin films were prepared using RF magnetron co-sputtering from 10 cm diameter ZnO and MgO targets on rotating SLG substrates at room temperature. The SLG is 15 cm × 15 cm in size and is located ~20 cm from each of the two targets. Sputtering is performed in pure Ar gas at a flow rate of 23 standard cm^3^/min and a pressure of 4 × 10^−3^ Torr, yielding a deposition rate of ~9.1 nm/min. Table 1 shows the pairs of RF power settings applied to the individual MgO and ZnO targets. These power settings enable control of the Mg atomic fraction *x* in the set of MZO samples over the range 0 ≤ *x* ≤ 0.42 as determined by EDS measurements. The as-deposited MZO samples were subjected to the thermal cycle of solar cell deposition, which involves annealing under vacuum at 250 °C for 120 min.

An X-ray diffractometer (Rigaku Ultima III, Rigaku Corporation, Akishima-shi, Tokyo, Japan) operating at 40 kV using Cu Kα (0.154056 nm) was applied to characterize the phase and crystallographic structure of the annealed samples as well as to estimate the lower limit of the crystalline grain size, the latter from the widths of the diffraction features. Following a procedure of baseline correction, the diffraction features are fitted to Lorentzian lineshapes using a Savitzky-Golay algorithm. The software package JADE^®^ (version 10.0, Materials Data, Inc., Livermore, CA, USA), used in conjunction with the diffractometer, enables isolation of instrumental broadening and its correction. The size of the coherently scattering domains in the MZO samples are on the scale of nanometers; however, due to the low deposition and annealing temperatures, the broadening related to the sample structure is much greater than the instrumental broadening.

The composition *x* of each annealed MZO film is determined by EDS using a scanning electron microscope (SEM) (Hitachi S-4800, Hitachi, Tokyo, Japan). To determine the standard deviation in composition for a given sample which defines the measurement error, this measurement is repeated five times at different locations.

Additionally, the annealed samples of this set were measured by NIR-UV-SE employing a rotating-compensator multichannel ellipsometer (Model M2000-DI, J. A. Woollam Co., Inc., Lincoln, NE, USA) for a photon energy range from 0.73 to 6.5 eV. The resulting data are analyzed to produce a parametric description of the complex dielectric function spectra as a function of a single parameter, the bandgap *E_g_*, appropriate for future applications of the description for as-deposited solar cell structures on which CdS/CdTe solar cells are fabricated. The parameterization is performed in terms of bandgap rather than composition due to the much smaller confidence limits on the bandgap measurement. The composition can be deduced from the bandgap by inverting the relationship to be presented in Section 3.2. To obtain the parametric description, the Levenberg-Marquardt algorithm is used for non-linear least-squares regression with photon energy independent free parameters. The goal of the analysis is to identify the set of parameters that minimize the error function between the experimental data and the simulated results and determine the confidence limits on these best fitting parameters. As shown in Table 1, this analysis allows us to conclude that the Mg atomic fraction range of 0 ≤ *x* ≤ 0.42 leads to a bandgap range of 3.264 eV ≤ *E_g_* ≤ 3.826 eV.

## 3. Results and Discussion

### 3.1. X-ray Diffraction (XRD)

**Figure 1:**
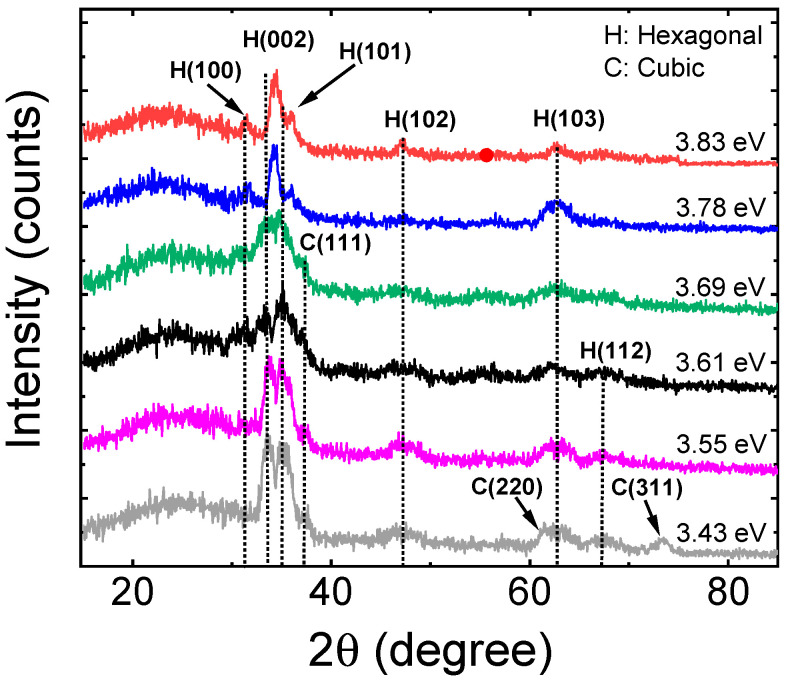
X-ray diffraction patterns measured with Cu-Kα radiation (wavelength: 0.154056 nm) for Mg*_x_*Zn_1−*x*_O thin films deposited with different Mg contents on room temperature soda lime glass substrates and annealed at 250 °C for 120 min. The samples are labelled according to their bandgaps.

Figure 1 shows the XRD patterns of six MZO thin films deposited on SLG substrates and annealed at 250 °C for 120 min, identified according to the bandgap values also provided in Table 1. In general, the weakness of the diffraction patterns indicates poor crystalline quality of the MZO films [29]. This may be attributed to the low deposition and annealing temperatures and the amorphous character of the underlying SLG substrate, as well as the relative thinness of the layers [30,31]. As a result, a thermodynamically metastable structure consisting of fine grained MZO evolves on the substrate due to the limited surface diffusion and bulk relaxation. Amorphous SLG was adopted as the substrate for the XRD analyses since only MZO crystalline features appear in the diffraction patterns whereas, for the conventional SnO_2_:F transparent conducting oxide coated substrates used in CdTe PV devices, SnO_2_ features will occur as well. In fact, most researchers studying PV applications of MZO have deposited these films on TEC™ glass, a SnO_2_:F coated product designed for various applications including PV (NSG Pilkington NA) [29,32,33]. For low temperature MZO deposition and annealing, however, the diffraction features of the SnO_2_:F layer of the TEC™ glass materials dominate the XRD pattern, making it more difficult to resolve the peaks associated with the MZO film.

The most clearly observable feature in the pattern for MZO on SLG in Figure 1 is near 34°, indexed as a (002) plane diffraction, revealing that the MZO films crystallize in the hexagonal crystal system. The presence of this feature also reveals that the dominant phase for all MZO films exhibits the wurtzite crystal structure which is the stable phase of ZnO [19,29,30,31,32,33,34,35,36]. With increasing Mg atomic fraction *x*, this (002) peak shifts to higher diffraction angle characteristic of the decrease in lattice parameter *c* due to the substitution of Mg at Zn lattice sites [29,30,31,33,34,36]. These results are in reasonable agreement with Wang et al. and Baines et al. who reported results on MZO films with Mg contents ranging from *x* = 0.0 to 0.25 deposited at higher substrate temperatures to different thicknesses [29,33]. The XRD pattern in Figure 1 shows additional weakly diffracting features also attributed to the wurtzite phase of MZO, the low index (100), (101), (102), and (103) diffraction peaks being recognizable. As noted, the weak diffractions are likely to occur due to the relatively low deposition and annealing temperatures used for this set of films and the thin layer deposition on an amorphous substrate [30]. Diffractions from (110) and (112) planes are also expected for the wurtzite crystal structure; however, these diffractions are too weak to be observed here. A literature study suggests that, in order for these peaks to be observed clearly, the MZO must be annealed at temperatures greater than 400 °C [31].

**Figure 2:**
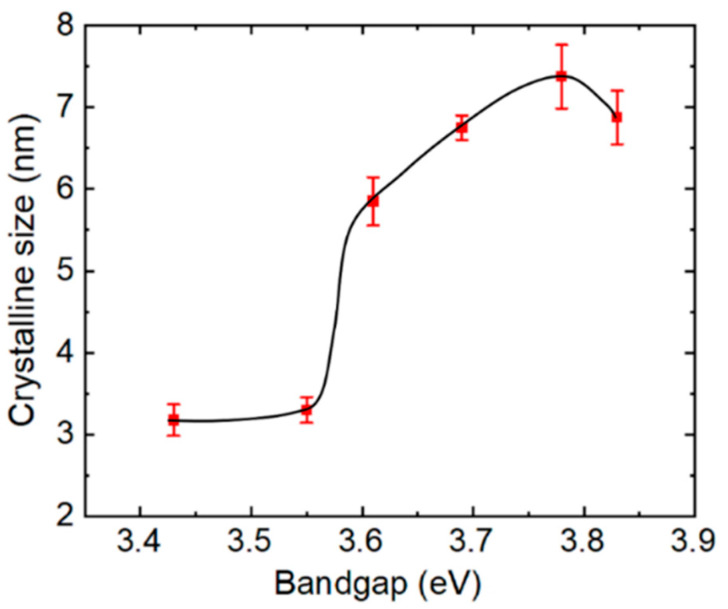
Crystalline size applying the Scherrer equation to the widths of the diffraction peaks for the MZO samples of Figure 1. The error bars are the standard deviations arising from independent measurements from the (002), (101), (102), and (103) diffractions, the four most visible peaks in Figure 1. The line is a guide for the eye.

Figure 2 shows the results of calculations of crystalline size for the samples of Figure 1 determined from the widths of the diffraction peaks applying the Scherrer equation. The standard deviations arise from independent determinations from the (002), (101), (102), and (103) peaks, the four most visible diffractions in Figure 1. Because the Scherrer equation describes the average sizes of coherently scattering crystalline units in the sample, it represents a lower bound on the crystallite size due to the presence of intragranular disorder due to alloying, point and planar defects, as well as variations in strain which also contribute to peak broadening. The defective nature of the crystallites as a result of the low deposition and annealing temperatures of the MZO may contribute to the small sizes over the range from 3 to 8 nm in Figure 2. It is interesting that the largest lower limit on the crystalline size occurs for the higher *x* compositional range of ~0.3–0.4, corresponding to a bandgap range of 3.7–3.8 eV, suggesting a reduction in intragranular defect density or grain size with the increase in *x*. Similar observations have been made in previous studies of low temperature magnetron sputtered MZO films [37,38]. This improvement in crystallinity with *x* may occur due to relaxation of the structure made possible by the substitution of Mg at Zn lattice sites.

Diffractions associated with a separate cubic rocksalt phase including those of the (111), (220), and (311) planes appear only for samples having Mg content within the range of 0 < *x* ≤ 0.28. These diffractions surprisingly disappear for samples with *x* > 0.28. Given the similarity of the lattice constants of rocksalt MgO and ZnO, it is possible that small Mg additions stabilize a Mg_1__−*x*_Zn*_x_*O cubic rocksalt phase in the nanocrystalline form of MZO resulting from the low temperature processing [39].

Finally, it should be emphasized that in order to avoid the low crystalline quality and secondary metastable phases, a possible strategy is to adopt elevated temperatures for deposition or post-deposition annealing. This strategy was found not only to improve film structure but simultaneously to widen the MZO film bandgap [30,34,35]. Such an approach, however, adds another parameter in addition to the ZnO and MgO target power levels that would require investigation of its effect on film composition.

### 3.2. Energy Dispersive X-ray Spectroscopy (EDS)

**Figure 3:**
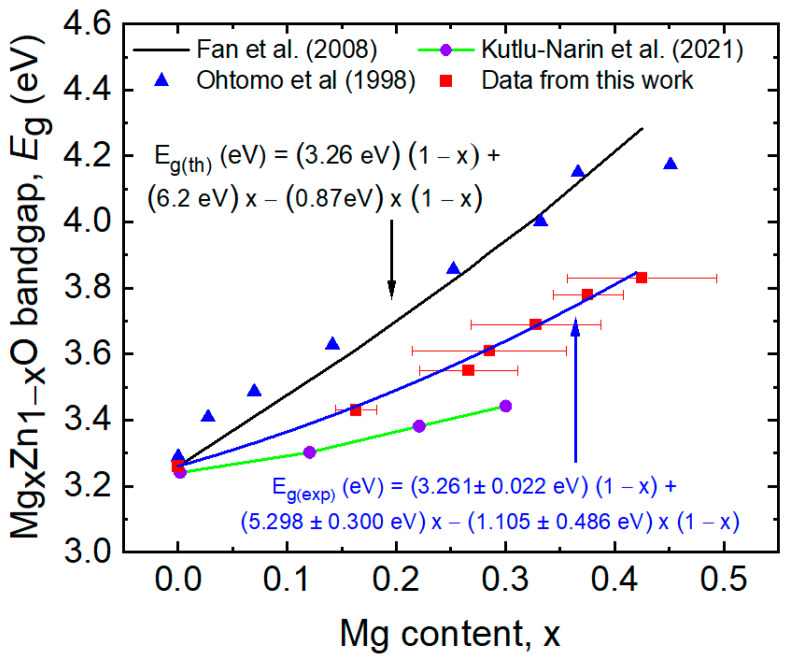
Bandgap of MZO thin films as determined from NIR-UV spectroscopic ellipsometry (SE) analysis plotted as a function of Mg atomic fraction as determined by energy dispersive X-ray spectroscopy (EDS) (squares). Over this range of Mg content, the MZO films exhibit a dominant wurtzite phase. Error bars on the Mg content arise from the standard deviation of multiple measurements whereas error bars on the bandgap arise from the confidence limits in the SE analysis. The latter are in the range of ±0.001 to ±0.003 eV, smaller than the data point size. The solid blue line is the best fitting quadratic expression. Experimental data from Refs. [16] (triangles) and [40] (circles) and a theoretical result from Ref. [41] incorporating a bowing parameter (black line) are also shown.

EDS measurements performed on the MZO films on SLG substrates listed in Table 1 have revealed an average oxygen content of 50.8 at.% with a standard deviation of 3.0 at.% for the set of seven samples. For each sample, multiple measurements at different locations led to standard deviations ranging from 0.4 to 2.5 at.%. Figure 3 shows the Mg atomic fraction *x* in MZO from EDS and bandgap *E_g_* from NIR-UV-SE, both also reported in Table 1. The bandgap values in Table 1 and Figure 3 are obtained from the dielectric function parameterization to be described in the next sub-section. Experimental results are presented in Figure 3 from Refs. [16,40] as well, and theoretical results are presented from Ref. [41] including the bowing effect but shifted by a constant energy to match the 3.26 eV bandgap of ZnO.

The reason for selecting the compositional range of *x* = 0 to 0.42 in this study is to span the range over which wurtzite is reported as the energetically favorable crystal structure and to avoid a dominant rocksalt phase that develops above *x* ≈ 0.5, depending on the deposition method and conditions [41,42,43]. It was found that an increase in substrate temperature can increase the Mg content of the single-phase MZO, and thus increase its bandgap, through the preferential desorption of Zn atoms [42,44,45]. Because the samples of Table 1 and Figure 3 were deposited at room temperature and annealed at 250 °C, any such enhancement in Mg concentration is avoided [16]. In this study in fact, the power applied to the MgO target is a factor of 2.5 times the power applied to the ZnO target to reach *x* = 0.42.

In Table 1 and Figure 3, the bandgap of the dominantly wurtzite MZO films is observed to increase from 3.264 to 3.826 eV as the Mg atomic fraction increases from *x* = 0 to 0.42 in accordance with a quadratic relationship. The relatively large error bars on the Mg content represent the standard deviations from multiple measurements at the different locations on the sample which also lead to bandgap variations over the surface that can be evaluated rapidly and with millimeter scale resolution using mapping SE methods similar to those described elsewhere for CdS and CdSe [8]. Within the resulting large error bars on the bowing parameter, this parameter agrees with the deduced theoretical value [41]. Our results for the best fitting relationship between *E_g_* and *x* lie within the range of those of Wang et al., Zhang et al., and Kutlu-Narin et al., who fabricated MZO thin films in low temperature co-sputtering and ultrasonic spray processes [29,37,40]. The differences among the various studies may be attributed to the influence exerted on the bandgap by film properties other than composition such as the film density, crystallinity, stress, and the concentration of *n*-type defects. For sputtered samples, differences in these properties may result from differences in target power levels, gas pressure, and substrate temperature.

### 3.3. Parameterization of the Mg_x_Zn_1−x_O Complex Dielectric Function Spectra versus Mg Content x

**Figure 4:**
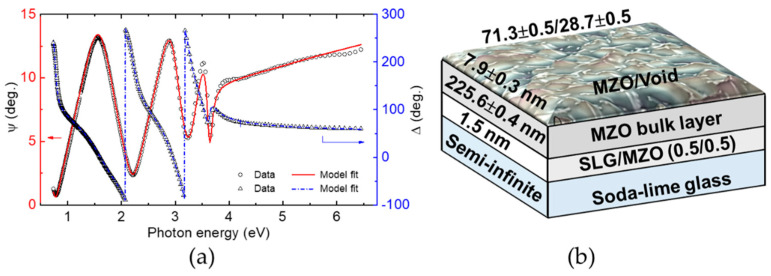
(**a**) Ellipsometric spectra (*ψ*, Δ) for a Mg*_x_*Zn_1−*x*_O sample fabricated on a soda lime glass substrate with power levels of 240 W and 500 W applied to the ZnO and MgO targets, respectively, yielding a Mg content of *x* = 0.32 ± 0.06 and a bandgap of *E*_0_ = *E_g_* = 3.690 ± 0.002 eV. The experimental data (points) are plotted along with the best fitting simulation (lines). The plot corresponds to results for a single representative location with coordinates (3.75, 4.69) (in cm), measured such that the center of the 15 cm × 15 cm sample is (0, 0). Here the Mg*_x_*Zn_1−*x*_O bulk layer and effective thicknesses are ~225.6 nm and 232.0 nm, respectively. The parameters defining the complex dielectric function spectra from the optical model appear in Figure 5 with best polynomial fits versus *E*_0_ = *E_g_* reported in Table 2. (**b**) The structural model and associated parameters corresponding to the best fit in (**a**) are shown.

Figure 4 shows ellipsometry spectra (*ψ*, Δ) measured from the film side of a sample consisting of thin film MZO deposited on SLG. The sample was prepared with a power level of 240 W applied to the ZnO target and 500 W applied to the MgO target (see Table 1), yielding *x* ~ 0.32. The intended effective thickness of the MZO film at the center of the sample was 250 nm. Layers approximately a factor of two thinner are used in device structures. The larger thickness enables higher sensitivity for analysis of the crystal structure, for the extraction of the MZO complex dielectric function spectra ε = ε_1_ − *i*ε_2_, and for evaluation of the depth uniformity of ε through the quality of the fit to the interference oscillations in the SE data. The pair of spectra in Figure 4 was obtained at a single location on the SLG/MZO sample with coordinates (3.75, 4.69) (in cm), measured such that the center of the 15 cm × 15 cm sample is (0, 0). The measurement location is relevant to mapping SE studies that will be described in future reports.

In the analysis of the (*ψ*, Δ) data set for each of the SLG/MZO samples, such as the set of Figure 4a, the spectra in ε to be used in a parameterization versus *x* must be determined together with the structural parameters. Furthermore, the dielectric function of the uncoated SLG substrate is also needed. These latter results were determined from an analysis of corresponding SE data acquired on a representative substrate before MZO deposition. Parameterization of ε = ε_1_ − *i*ε_2_ for the MZO layers applied an analytical expression consisting of the combination of a constant offset to the real part of the dielectric function ε_1_ and two critical point (CP) oscillators having a complex analytical form. Each CP oscillator is derived assuming parabolic bands when the band energies are plotted versus wave vector. A lower energy oscillator simulates the bandgap features in ε_1_ and ε_2_ whereas a higher energy feature is outside the measured spectral range and serves to generate dispersion in ε_1_ over the measured range. The non-zero, but weak ε_2_ spectrum below the lowest CP energy or bandgap *E_g_*, representing broadening of the bandgap transition from various sources, cannot be described accurately using the CP oscillator expression. Thus, for energies below an assigned near-bandgap transition energy, i.e., *E* < *E_t_*, the CP generated expression for ε_2_ is replaced by an Urbach absorption tail. The associated weak contribution of this tail to ε_1_ through Kramers-Kronig relations is neglected. The SE analysis procedure and the representative results in Figure 4a for the SLG/MZO sample with *x* ~ 0.32 and *E*_0_ = *E_g_* = 3.690 ± 0.002 eV will be described in detail in the following paragraphs.

The final best fit to the experimental data in Figure 4a applies the three-parameter structural model of Figure 4b that includes MZO bulk and surface roughness layer thicknesses along with the MZO volume percentage in the surface roughness layer. The 1.5 nm SLG/MZO interface layer is established in a measurement of the uncoated SLG substrate and is interpreted as surface modulations on the glass that are filled by MZO upon its deposition on the SLG. The dielectric functions of the interface modulation layer and the MZO surface roughness layer are determined using the Bruggeman effective medium approximation (EMA) [46]. This same structural model is used for all SLG/MZO samples.

The development of the appropriate optical model that can be used for all samples is a greater challenge. Given that the dielectric function is modeled with a constant real part, two CP oscillators, and an Urbach tail, as noted above, there are a maximum of 13 possible optical property free parameters. The mathematical expression applied for ε_1_ over the full range of photon energy *E* and for ε_2_ over the range of *E* above *E_t_*, the Urbach to band-to-band transition energy, is given by [47,48]

(1) $$\varepsilon \left(E\right)=\varepsilon_{1}\left(E\right)-i\varepsilon_{2}\left(E\right)=\varepsilon_{1o}+\sum_{n}A_{n}\left\{\exp\left(i\varphi_{n}\right)\right\}{\left\{\left(\Gamma_{n}/2\right)/[E_{n}-E-i\left(\Gamma_{n}/2\right)]\right\}}^{\mu_{n}}.$$

where *A_n_*, *E_n_*, Γ*_n_*, *φ_n_*, and *μ_n_* are the amplitude, resonance energy, broadening energy, phase, and exponent, respectively, for the CP indexed by *n* (*n* = 0, 1), and ε_1*o*_ is the real constant contribution to ε_1_. Thus, of the maximum 13 parameters, 10 are associated with the two CP oscillators. One parameter is the constant contribution to ε_1_, and two define an expression for ε_2_ describing the Urbach tail for *E* < *E_t_*, which takes the form:

(2) $$\varepsilon_{2}\left(E\right)=\varepsilon_{2}\left(E_{t}\right)\exp\left[\left(E-E_{t}\right)/E_{u}\right];\;E<E_{t},$$

where *E_u_* is the Urbach parameter and *E_t_* is the transition energy. This Urbach tail expression is used to replace ε_2_ from Equation (1) for *E* < *E_t_*. In this model, *E_t_* was set equal to the fundamental bandgap *E*_0_ which is the critical point energy; thus *E_t_* = *E*_0_ = *E_g_* [49]. For these MZO samples no deep sub-bandgap absorption component was detected in ε_2_, as was observed in some CdS thin films fabricated by sputtering as described in Ref. [8].

Because the higher energy CP lies above the spectral range of measurement, it was not possible to vary all 13 parameters of the optical model for any given sample. In fact, it was necessary to fix the resonance energy *E*_1_ of the higher energy CP to prevent instability of the fitting and strong correlations between pairs of parameters of this CP. The fixed value of *E*_1_ = 8.754 eV was chosen for consistency with the results of Schmidt-Grund et al., who measured the ordinary dielectric function of *c*-plane MZO thin films by SE over the photon energy range from 4.5 to 9.5 eV [50]. With *E*_1_ fixed and *E_t_* linked to *E*_0_, as described in the previous paragraph, the correlations become manageable such that a fit to the (*ψ*, Δ) spectra is possible for the pure ZnO sample (*x* = 0) with the lowest bandgap of 3.264 eV. Thus, the data for ZnO could then be fit with a total of three structural and 11 optical parameters. Because the CP of interest is that at lower energy near the center of the measured spectral range, its parameters of energy, amplitude, and broadening which control the position, strength, and width of the bandgap feature, respectively, are not significantly correlated in this fit of the data for the pure ZnO sample. The two parameters of exponent and phase that control the detailed shape of this CP, however, are more strongly correlated. Similarly, the lower energy CP amplitude and the constant contribution ε_1*o*_ are also correlated. As a result, for analysis of the set of MZO alloys used in dielectric function parameterization, the phase *φ*_0_ of the lower energy CP and the real contribution ε_1*o*_ were fixed at (−6.613°, 1.729), respectively, values obtained in the fit to the (*ψ*, Δ) spectra for the pure ZnO sample.

The analysis of the pure ZnO sample thus led to a 12-parameter model with three structural and nine optical free parameters in an initial analysis of the set of MZO samples. As expected, relatively large correlations are still observed in this analysis between pairs of the remaining four parameters of the higher energy CP. Such correlations warrant fixing these parameters as (*A*_1_, Γ_1_, *φ*_1_, *μ*_1_) = (3.902, 0.0248 eV, 113.156°, 0.392), average values obtained from the set of MZO samples. Then, in a final iteration of the fitting procedure applied to the (*ψ*, Δ) spectra for the set of MZO samples deposited on SLG, the three structural free parameters and the five remaining optical free parameters are determined along with their confidence limits. The latter parameters include the lower energy CP amplitude *A*_0_, resonance energy *E*_0_ = *E_g_*, broadening Γ_0_, and exponent *μ*_0_, as well as the Urbach energy *E_u_*. In this final iteration, higher fitting stability with no significant reduction in the quality of the fits for the collection of samples could be obtained. By using this five-parameter optical model, the confidence limits on the resulting parameters and the fluctuations were sufficiently small that well-defined trends versus *x* could be identified. In fact, the use of five optical free parameters to describe ε = ε_1_ − *i*ε_2_ for the set of MZO layers is understandable as a general consequence of the spectral range and photon energy locations of the two CPs of MZO. Similar two CP approaches were used for other transparent conducting oxide materials and HRTs [49,51].

The solid lines in Figure 4a provide the final best fitting results for the MZO sample with *x* = 0.32 using the eight free parameter model whereby the optical model was reduced to five free parameters as described in the previous paragraph. Figure 4b shows the three best fitting structural parameters from this model including the bulk and surface roughness layer thicknesses of 225.6 ± 0.4 nm and 7.9 ± 0.3 nm, respectively. Application of the Bruggeman EMA led to a best fitting MZO content of 71.3 ± 0.5 vol.% in the roughness layer. Because the roughness layer thicknesses for all MZO samples are more than an order of magnitude smaller than the wavelength of the SE probe light within the material, even for the shortest probe wavelengths, then we expect that the EMA approach is valid and the structural analysis is accurate [52]. Previous studies of amorphous semiconductors, transparent conducting oxides, and metals have demonstrated close linear correlations between the roughness thickness deduced by atomic force microscopy and that by SE over the SE roughness thickness range of 1–10 nm. These previous results also support the validity of structural analyses by SE for < 10 nm thick roughness layers [53,54].

**Figure 5:**
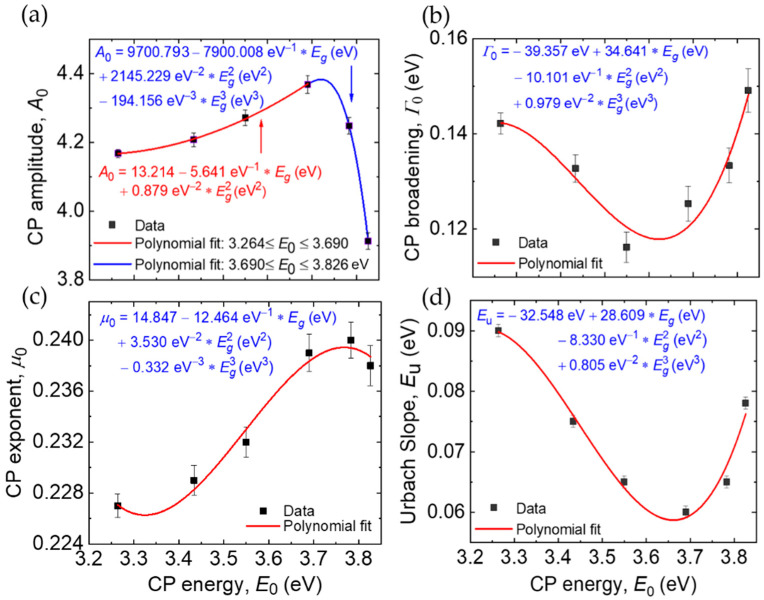
Best fitting variable parameters of lower energy CP (**a**) amplitude, (**b**) broadening, and (**c**) exponent, as well as the (**d**) Urbach slope representing the MZO dielectric function according to Equations (1) and (2).

**Figure 6:**
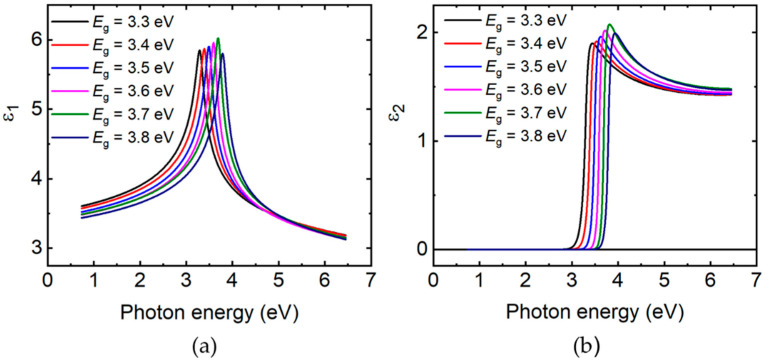
Analytically determined complex dielectric function (ε = ε_1_ − *i*ε_2_) spectra for hypothetical Mg*_x_*Zn_1−*x*_O films with different Mg contents *x* and bandgap energies *E*_0_ = *E_g_*. (**a**) photon energy of ε_1_; (**b**) photon energy of ε_2_. These spectra were determined from Equations (1) and (2) using the bandgap and linked *E_t_* value, seven fixed parameters, and four polynomial descriptions as presented in Table 2. The polynomial expressions in Table 2 enable linking the additional four parameters to the bandgap, enabling the dielectric function spectra to be specified by the single bandgap variable.

Figure 5 shows the four variable optical parameters and confidence limits including the bandgap CP parameters *A*_0_, Γ_0_, and *μ*_0_, along with the Urbach energy *E_u_*, all plotted as functions of the fifth variable parameter which is the bandgap energy *E*_0_ = *E_g_*. The bandgap is used in the plot as it is more accurately defined than the composition obtained from EDS analysis, as noted previously. Table 2 shows the best fitting polynomial expressions for the four variable parameters from the regression analyses, given in terms of *E*_0_ = *E_g_*. Table 2 also shows the fixed ε_1*o*_ value and the six fixed oscillator parameters. In fact, Figure 5 and Table 2 show that although *A*_0_, Γ_0_, *μ*_0_, and *E_u_* are assumed to be independent variables in the analysis, their values appear closely linked to the *E*_0_ = *E_g_* value as indicated by the relatively smooth variations in Figure 5. Thus, the polynomials as well as the linked and fixed parameters in Table 2 in conjunction with Equations (1) and (2) provide analytical expressions for the dielectric function over the spectral range from 0.73 to 6.5 eV for any specified value of the MZO bandgap over the range from *E_g_* = 3.264 eV for *x* = 0 to *E_g_* = 3.826 eV for *x* = 0.42. Figure 6 shows the analytical forms of the dielectric functions of six hypothetical samples with bandgaps ranging from 3.30 to 3.80 eV in steps of 0.1 eV. These spectra are generated by Equations (1) and (2) and the parameters reported in Table 2.

The four best fitting polynomials of Figure 5 show consistent extrema in the range *E_g_* = 3.65–3.75 eV, corresponding to Mg atomic fractions of *x* ~ 0.30–0.35. These include maxima in *A*_0_ and *μ*_0_ near *E_g_* ~ 3.75 eV and minima in Γ_0_ and *E_u_* near *E_g_* ~ 3.65 eV. In Figure 5a, the bandgap CP amplitude *A*_0_ is observed to increase with increasing Mg content *x* for *x* < 0.35, reaching its maximum near *x* = 0.35, and then to decrease for the highest *x* values. The decrease in *A*_0_ suggests a structural transition for *x* > 0.35 associated with an abrupt increase in void content within the MZO. The XRD indicates a dominant wurtzite phase both above and below this *x* = 0.35 transition, and therefore no change in the crystal structure. The widths of the XRD peaks indicate an improved crystalline structure or larger grain size in the compositional range where the extrema in the optical parameters occur in comparison with the lower *x* (<0.25) samples.

As the CP amplitude increases with *x* for *x* < 0.30, both the bandgap CP broadening parameter and the Urbach tail slope are observed to decrease, reaching consistent minima near *x* = 0.31 as shown in Figure 5b,d. The broadening parameter associated with optical transitions in general, as well as the Urbach energy associated with bandgap transitions, are well known to be very sensitive to the structure of the material. Considering materials of the same composition, as exemplified by pure silicon, the single crystal shows a lower broadening parameter than polycrystalline and nanocrystalline thin films, which in turn show lower broadening parameters than amorphous thin films [55]. The introduction of various structural features can account for these broadening trends and for the tailing of the absorption onset into the bandgap. These include grain boundaries, intragranular defects, and strain in crystalline films, as well as disorder, density variations, and voids in amorphous films. Relevant for the case of MZO, alloying resulting in a disordered solid solution may also increase broadening and band tailing in crystalline and amorphous materials [56].

These free parameters are plotted versus the bandgap CP energy *E*_0_ = *E_g_* and are obtained along with *E*_0_ in regression analyses of SE data acquired on selected SLG/MZO samples. A three-parameter structural model as in Figure 4b and a five-parameter optical model are used in the analysis. The solid lines depict best fitting polynomials that describe the four parameters continuously versus *E*_0_ = *E_g_*. The polynomials are presented in Table 2. The necessity of a two-segment polynomial fit for the CP amplitude in panel (a) indicates a structural transition with increasing *x* near *x* ~ 0.35 and *E*_0_ = *E_g_* ~ 3.75 eV. The second polynomial segment is constrained so that the amplitudes and the first derivatives of the amplitudes with respect to *E*_0_ are continuous at the transition bandgap of 3.690 eV. Minima in both the bandgap CP broadening and Urbach slope with increasing *x* near *x* ~ 0.3 and *E*_0_ = *E_g_* ~ 3.65 eV suggest a minimum in the density of defects that scatter electrons or a maximum in the crystalline grain size just before the structural transition with increasing *x*.

The broadening parameter of optical transitions can also be understood as inversely proportional to an excited state lifetime or to an excited carrier mean free path, the latter providing information on the relative grain size of polycrystalline films or more generally the concentration of scattering centers. Thus, combining this general discussion with the experimental observations of Figure 5 suggests that the excited state lifetime and associated electron mean free path for excitations in the MZO samples increase with *x* at low *x* reaching a maximum at *x* = 0.31. This may suggest an increase in crystalline grain size or quality over the range from *x* = 0 to 0.31 with a reversal accompanying the structural transition for *x* > 0.31. Thus, one may conclude that MZO with *x* ~ 0.3 exhibits the lowest concentration of crystalline defects that act as scattering sites [49,55]. This result is surprising, given that alloying typically results in enhanced broadening and Urbach tails, but is qualitatively consistent with the XRD measurements of crystallinity, considering the larger error bars in Figure 2 relative to those on the optical parameters in Figure 5. A similar trend in *E_u_* to that in Figure 5 but at higher *x* was reported previously, however, and attributed to relaxation of the wurtzite structure by an incipient cubic phase [57].

Additional literature reports describe in detail the phase and structural transitions with increasing Mg content *x* in MZO starting from the pure wurtzite phase at low *x*. Such reports also present correlations of these transitions with crystalline quality. Using a pulsed laser deposition process and a *c*-plane sapphire substrate at a deposition temperature of 600 °C, Ohtomo et al. detected a transition in the MZO films with increasing Mg content *x* at a composition of *x* ~ 0.33 [16]. This was attributed to the incorporation of a (111) oriented MgO rocksalt phase impurity within the dominant wurtzite phase and suggested a solubility limit at least as high as *x* = 0.33 for MgO in ZnO under the deposition conditions used in their study. Applying first principles computations, Maznichenko et al. [43] predicted a phase transition from wurtzite to rocksalt at a Mg content of *x* = 0.33, consistent with the studies of Ref. [16]. In a laser deposition process for MZO similar to that of Ref. [16], using *c*-plane sapphire substrates held at a higher temperature of 750 °C, an increase in optical transition broadening with increasing Mg content was observed to the highest Mg contents of the study, *x* ~ 0.5 [50]. This increase was attributed to a decrease in structural homogeneity with Mg incorporation that also resulted in a damping of the bandgap excitonic features. The results reported in these studies are likely to represent behavior close to equilibrium given the high substrate temperatures.

Different behavior was observed for MZO films deposited by DC sputtering on CaF_2_ substrates at lower temperatures of 250 °C, as reported by Huso et al., who characterized the Urbach energy for films over the range from *x* = 0 to 0.68 [57]. For Mg contents *x* < 0.5, the dominant contribution to the Urbach tail was ascribed to grain boundaries and defects such as dangling bonds whereas for the highest Mg contents of *x* > 0.6, an inhomogeneous mixture of phases was a possible contributing factor. Additionally in Ref. [57], *E_u_* was found to exhibit a minimum similar to that of Figure 5, but at a higher Mg content of *x* ~ 0.55. This minimum in *E_u_* was observed prior to an abrupt increase versus *x*, and the minimum was attributed to incipient formation of the rocksalt phase which enables relaxation of the wurtzite structure and a reduction in band tail states [57]. A minimum in *E_u_* may be a common feature of low temperature sputtered films with substantial compressive stress. The incorporation of Mg, in addition to the proposed incipient rocksalt phase, may serve to relax compressive stress and account for the XRD trend in Figure 2 and the gradual reduction in *E_u_* in Figure 5 for *x* < 0.3, prior to the reversal for *x* > 0.35 that accompanies the structural transition. Although an enhancement of the rocksalt phase is not observed with increasing *x* for *x* > 0.35, a component below the detection limit may be present.

### 3.4. Future Applications of Dielectric Function Parameterization for Characterization of CdTe Device Structures

Currently, two important applications of this work to CdTe device structure and optical property analysis are being pursued. These applications stem naturally from the parameterization of the complex dielectric function ε for the MZO thin film materials that was presented and discussed in Section 3.3. In addition, an opportunity for further optical characterization of the MZO thin films in device configurations was identified through previous studies, and preliminary work in this direction was performed as well. Although the full details are beyond the scope of the present article, the experimental methodologies of the applications of MZO parameterization and the nature and goals of the opportunity for further optical characterization of MZO-containing device structures will be introduced in this sub-section.

In PV manufacturing, uniformity of the HRT layer over the area of the PV panel is critical. The application of MZO as an HRT layer introduces further challenges beyond those posed by the more conventional SnO_2_ high resistivity transparent layer. Not only must the thickness of the HRT layer be carefully controlled for uniformity over the module, but the Mg content, and thus the bandgap, must be similarly controlled in order to achieve uniformly high sub-cell performance. Mapping spectroscopic ellipsometry (M-SE) can be applied in order to characterize the uniformity in thickness and bandgap for the MZO layer when deposited on standard TEC™ glasses. As compared to the SLG/MZO structure characterized here, the three layers that coat the SLG substrate of TEC™ glass and the thicker roughness layer of the TEC™/MZO interface introduce greater complications in SE data analysis. Ideally the more complicated structural model for TEC™/MZO should include SnO_2_:F/MZO interface, MZO bulk, and MZO surface roughness layers. The capability of distinguishing these layers is of interest so that the effective thickness of the MZO can be accurately determined.

For structural model development within the scope of the proposed M-SE analysis of TEC™/MZO, it may be necessary to map pairs of corresponding locations on the PV panel both before MZO deposition on the TEC™ glass and after deposition and subsequent annealing. Analysis of the M-SE measurement before MZO deposition will provide maps of the thicknesses of the SnO_2_, SiO_2_, and SnO_2_:F bulk layer constituents of the TEC™ glass as well as a map of the SnO_2_:F surface roughness layer thickness. Then in the subsequent analysis of the M-SE data acquired after both the MZO deposition and the annealing step, it can be assumed that the MZO completely fills the void volume in the SnO_2_:F surface roughness layer at the top of the TEC™ glass multilayer structure and that the SnO_2_:F roughness layer is then converted to an equivalent thickness SnO_2_:F/MZO interface layer. Once the MZO fills this layer, it is difficult to identify this layer and determine its thickness independently of the adjoining SnO_2_:F and MZO bulk layers as a result of the similarity of the three sets of ε spectra. This leads to an ill-defined interface and greater correlations among the deduced thicknesses of the SnO_2_:F and MZO bulk layers and the SnO_2_:F/MZO interface layer. Thus, for the final TEC™/MZO structural model in the M-SE data analysis, the SnO_2_:F/MZO roughness layer may be fixed at the deduced SnO_2_:F layer roughness thickness obtained prior to MZO deposition. Only by applying this procedure may it be possible to extract with confidence the remaining MZO bulk and surface roughness layer thicknesses and hence the effective thickness.

Beyond M-SE characterization, through-the-glass spectroscopic ellipsometry (TG-SE) in the near-IR to near-UV spectral range can be performed as another application of the ε parameterization of Section 3.3, in this case for the metrology of complete CdTe solar cell device structures incorporating MZO as an HRT layer. Furthermore, TG-SE and M-SE data collection modes can be combined for analysis of completed PV panels. In the TG-SE application, parametric models for ε versus photon energy are desired for all components of the complete CdTe device structure of TEC™/MZO/CdS/CdTe/back-contact, especially for the components whose optical properties may vary from deposition to deposition or depend on the underlying material. Such models have been developed and described in Refs. [49,58] for all components with the exception of the MZO HRT layer, whose parametric expressions versus composition *E_g_* were presented in Section 3.3.

Because of the complexity of the complete multilayer structure of the CdTe solar cell, a systematic step-by-step procedure is required for TG-SE data analysis of the TEC™/MZO/CdS/CdTe/back-contact structure. In this analysis, a two-stage hierarchy of the most important structural and optical property parameters is established as described in Ref. [51]. The structural and optical models used as the starting point of this analysis are based on four assumptions. (i) The nominal or intended thicknesses are adopted for all layers including, from the SLG to the back of the cell structure, SnO_2_ (20 nm), SiO_2_ (30 nm), SnO_2_:F (330 nm, for TEC™-15), MZO (100 nm), CdS (50 nm), and CdTe (2000 nm). (ii) Fixed spectra in ε_1_ and ε_2_ are used from the existing database including those for MZO established based on the intended composition in the fabrication process [49,58]. (iii) No interface or back surface roughness layers or structural non-idealities, such as thickness non-uniformity, are used in the starting structural model, and (iv) no optical non-idealities such as stress-induced birefringence in the glass are used in the associated optical model. The starting point for the introduction of an interface roughness layer into the structural model is a 50/50 vol.% mixture applying the Bruggeman EMA [46]. In the second stage of the hierarchy in which the optical properties are varied, the composition of the interface layer can be varied. This overall approach has been successfully applied in CdTe PV metrology as described in Ref. [51]. As in the M-SE studies, the goal is to extract the MZO effective thickness and bandgap from complete or nearly complete device structures.

Finally, an additional means of characterizing the optical properties of MZO-containing device structures is expected to be informative toward the refinement of the M-SE and TG-SE models. In previous studies of CdTe device structures incorporating SnO_2_:F as the transparent conducting oxide and SnO_2_ as the HRT layer, infrared spectroscopic ellipsometry (IR-SE) measurements were performed at different stages of fabrication to gain insights into the interaction between adjoining layers [59,60]. In these studies, the role of CdS deposition in modifying the underlying SnO_2_:F free electron and defect properties and the role of the intervening SnO_2_ in suppressing these interactions can be characterized. In addition, the free electron concentration (or an upper limit for it, if below ~10^18^ cm^−3^) can be determined for the HRT layer along with how it may change upon interaction with the underlying SnO_2_:F or upon over-deposition of CdS. When applied to MZO serving as the HRT layer, such studies are critically important in understanding the performance of the MZO material as it exists as a layer in the device structure.

Future publications will report the applications of M-SE and TG-SE to CdTe device structure analyses that are facilitated by the complex dielectric function parameterization for MZO presented in Section 3.3. The results of studies of MZO layer interactions in devices enabled by IR-SE characterization will also be reported in future publications.

## 4. Conclusions

MZO thin films ~250 nm thick with Mg contents *x* spanning the range of 0 ≤ *x* ≤ 0.42 were fabricated by co-sputtering of MgO and ZnO targets on room temperature soda lime glass (SLG) substrates for applications in CdTe photovoltaic device structures. After deposition, the MZO layers were subjected to the thermal cycle of the CdTe deposition by sputtering which involves annealing the MZO to 250 °C for 120 min. The focus of this research on MZO is the development of spectroscopic ellipsometry (SE) first for determination and parameterization of the complex dielectric function and in the future for application of the resulting database in advanced optical metrology after the different stages of photovoltaic device fabrication. X-ray diffraction patterns of the annealed MZO films on SLG reveal broad peaks over the full range of 0 ≤ *x* ≤ 0.42, and thus poor crystallinity, with a dominant wurtzite phase which is the stable phase of ZnO. The deduced lattice parameter *c* decreases continuously with increasing Mg content, indicative of a substitutional solid solution of ZnO and MgO. Diffractions associated with a separate cubic rocksalt phase, the stable phase of MgO, appear only for samples with 0 < *x* ≤ 0.28. One possible explanation is that low Mg additions stabilize a Mg*_x_*Zn_1−*x*_O cubic rocksalt phase in the nanocrystalline structure that results from the sputter deposition process at room temperature [39]. Along with the loss of this cubic phase, the XRD peaks tend to sharpen with increasing *x* suggesting a decrease in the concentration of intragrain defects or an increase in crystallite size, following the same general trend as observed for the width of the bandgap optical CP feature and the Urbach tail.

For each of the MZO layers on SLG, the real and imaginary parts of the complex dielectric function ε = ε_1_ − *i*ε_2_ were determined from an analysis of spectroscopic ellipsometry data using a three-layer structural model consisting of SLG/interface/bulk/(surface roughness). This structural model incorporates three free parameters, the MZO bulk and surface roughness layer thicknesses and the surface roughness layer MZO volume percentage. The optical model for the bulk layer MZO component of the sample was developed using analytical expressions for ε that incorporate photon energy independent fitting parameters. These expressions include the combination of two CP oscillators, a constant contribution to ε_1_, and an Urbach tail contribution to ε_2_, the latter starting from below the bandgap and increasing exponentially with increasing photon energy up to the bandgap. Thus, a potential total of 13 free parameters are used to describe ε_1_ and ε_2_. The two CP oscillators of the MZO bulk layer represent the E_0_ bandgap whose value ranges from 3.26 to 3.83 eV for *x* increasing from 0 to 0.42 and the E_1_ transitions with a resonance energy assumed to be fixed with *x*. By first focusing on the *x* = 0 sample and then iterating the fitting procedure for the set of MZO samples, the number of optical free parameters describing the MZO ε spectra can be reduced to five, including the E_0_ CP amplitude, resonance energy or bandgap, broadening, and exponent, as well as the Urbach tail slope or Urbach energy. Thus, the final model including both the structural and optical components incorporates eight variable parameters in all.

The most critical of the five variable optical parameters in the metrology of MZO is the bandgap, i.e., the resonance energy of the first CP, *E*_0_ = *E_g_*, which shows a smooth dependence on the composition consistent with a dominant wurtzite phase over the full range of *x* from 0 to 0.42 and a bowing parameter consistent with previous studies [41]. The other variable optical parameters describing the MZO show systematic trends with the bandgap and composition. As functions of *x*, both the CP broadening parameter and the Urbach slope show minima at a composition of *x* = 0.31, which corresponds to a bandgap of 3.65 eV, suggesting that at this composition and bandgap, the MZO exhibits either a minimum concentration of defects in the bulk of the crystallites or a maximum in the grain size in consistency with the XRD results. A reduction in non-uniform stresses within crystallites with increasing *x* over the range 0 < *x* ≤ 0.31 may also contribute to the broadening reductions in the optical and XRD features. A previous study has detected a similar minimum in the Urbach slope at intermediate *x* between zero and unity and attributed the effect to an incipient phase transition to rocksalt [57]. In the present study, the observation may be related to the high stress of low temperature sputtered films which is relaxed by the incorporation of Mg atoms. Gradual elimination of a rocksalt phase detected in the XRD may also lead to a minimum in the broadening and Urbach slope. At a higher composition of 0.35 and bandgap of 3.72 eV, the E_0_ CP amplitude drops abruptly indicating a structural transition to higher void content. This transition is accompanied by an increase in the E_0_ bandgap broadening parameter and Urbach slope above their minima possibly due to the structural defects associated with voids.

Important for MZO metrology is the fact that the variable optical parameters such as the E_0_ CP amplitude, broadening, and the exponent, as well as the Urbach slope exhibit smooth trends when plotted as functions of the MZO bandgap energy and so can be fit with polynomials. For the E_0_ CP amplitude, a two-segment polynomial fit is required to account for the abrupt structural transition at *E_g_* ~ 3.72 eV. As a result of the best fitting polynomial and analytical expressions, the ε_1_ and ε_2_ spectra for MZO can be determined from the specification of the bandgap, a single parameter, over the *E_g_* = 3.26 to 3.83 eV range, which in turn can be specified from the Mg content *x* over the *x* = 0 to 0.42 range. Thus, the ε_1_ and ε_2_ spectra can be generated for hypothetical MZO samples of any bandgap and associated composition over the available spectral and bandgap ranges. Such a capability is an important first step in the development of SE methods for analysis of complicated CdTe-based device structures that incorporate MZO as a high resistivity transparent layer. The goal of such SE metrologies is the determination of the effective thickness and bandgap of the MZO layer and the variations of these key characteristics with location over the area of a PV panel.

## Figures and Tables

**Table 1:** The RF magnetron power settings for sputtering of MgO and ZnO targets, the Mg molar fraction *x* for each sample as determined from energy dispersive X-ray spectroscopy measurements, and the resulting MZO bandgap from NIR-UV spectroscopic ellipsometry measurements. For the sample with the third highest *x*, the power applied to the MgO target has reached its limit, and for higher *x*, the power applied to the ZnO target is reduced.

ZnO RF Power (Watt)	MgO RF Power (Watt)	Mg Content, *x* (Atomic Fraction)	Mg*_x_*Zn_1−_*_x_*O *E*_0_ *= E_g_* (eV)
240	0	0	3.264 ± 0.001
240	200	0.16 ± 0.02	3.433 ± 0.002
240	300	0.26 ± 0.05	3.554 ± 0.003
240	400	0.28 ± 0.07	3.613 ± 0.002
240	500	0.32 ± 0.06	3.690 ± 0.002
225	500	0.37 ± 0.03	3.781 ± 0.003
200	500	0.42 ± 0.07	3.826 ± 0.002

**Table 2:** Polynomial expressions in terms of bandgap CP energy *E*_0_ = *E_g_* for the four variable optical parameters along with values for the single linked and seven fixed optical parameters deduced in NIR-UV spectroscopic ellipsometry analyses of SLG/MZO samples. The information in this table along with Equations (1) and (2) can be applied to predict the dielectric function of Mg*_x_*Zn_1−*x*_O for any specified value of *E_g_* within the range 3.264 eV ≤ *E_g_* ≤ 3.826 eV.

Oscillator	Parameter	Value/Expression in Terms of *E_g_*
*E*_0_	*A*_0_	13.214 − 5.6414*E_g_* + 0.879*E_g_*^2^ (3.264 eV ≤ *E_g_* ≤ 3.690 eV) 9700.7933 − 7900.0075*E_g_* + 2145.2291*E_g_*^2^ − 194.1559*E_g_*^3^ (3.690 eV ≤ *E_g_* ≤ 3.826 eV)
	*E*_0_ (eV)	*E_g_*
	Γ_0_ (eV)	−39.357 + 34.641*E_g_* − 10.101*E_g_*^2^ + 0.979*E_g_*^3^
	*φ*_0_ (degree)	−6.613
	*μ*_0_	14.847 − 12.464*E_g_* + 3.530*E_g_*^2^ − 0.332*E_g_*^3^
*E*_1_	*A*_1_	3.902
	*E*_1_ (eV)	8.754
	Γ_1_ (eV)	0.0248
	*φ*_1_ (degree)	113.156
	*μ*_1_	0.392
	ε_1*o*_	1.729
Urbach absorption tail	*E_t_* (eV)	*E_g_*
	*E_u_* (eV)	−32.548 + 28.609*E_g_* − 8.3303*E_g_*^2^ + 0.8054*E_g_*^3^

## Data Availability

The data presented in this study are available upon request from the corresponding author.

## References

[1] Green M., Dunlop E., Hohl-Ebinger J., Yoshita M., Kopidakis N., Hao X. (2021). Solar cell efficiency tables (version 57). Prog. Photovolt. Res. Appl..

[2] Gloeckler M., Sankin I., Zhao Z. (2013). CdTe solar cells at the threshold to 20% efficiency. IEEE J. Photovolt..

[3] Paudel N.R., Yan Y. (2014). Enhancing the photo-currents of CdTe thin-film solar cells in both short and long wavelength regions. Appl. Phys. Lett..

[4] Geisthardt R.M., Topič M., Sites J.R. (2015). Status and potential of CdTe solar-cell efficiency. IEEE J. Photovolt..

[5] Sites J., Munshi A., Kephart J., Swanson D., Sampath W.S. (2016). Progress and challenges with CdTe cell efficiency. Proceedings of the 2016 IEEE 43rd Photovoltaic Specialists Conference (PVSC).

[6] Kanevce A., Reese M.O., Barnes T.M., Jensen S.A., Metzger W.K. (2017). The roles of carrier concentration and interface, bulk, and grain-boundary recombination for 25% efficient CdTe solar cells. J. Appl. Phys..

[7] Metzger W.K., Grover S., Lu D., Colegrove E., Moseley J., Perkins C.L., Li X., Mallick R., Zhang W., Malik R. (2019). Exceeding 20% efficiency with in situ group V doping in polycrystalline CdTe solar cell. Nat. Energy.

[8] Razooqi Alaani M.A., Koirala P., Pradhan P., Phillips A.B., Podraza N.J., Heben M.J., Collins R.W. (2021). Tailoring the CdS/CdSe/CdTe multilayer structure for optimization of photovoltaic device performance guided by mapping spectroscopic ellipsometry. Sol. Energy Mater. Sol. Cells.

[9] Colegrove E., Banai R., Blissett C., Buurma C., Ellsworth J., Morley M., Barnes S., Gilmore C., Bergeson J.D., Dhere R. (2012). High-efficiency polycrystalline CdS/CdTe solar cells on buffered commercial TCO-coated glass. J. Electron. Mater..

[10] Song T., Kanevce A., Sites J.R. (2016). Emitter/absorber interface of CdTe solar cells. J. Appl. Phys..

[11] Ablekim T., Perkins C., Zheng X., Reich C., Swanson D., Colegrove E., Duenow J.N., Albin D., Nanayakkara S., Reese M.O. (2018). Tailoring MgZnO/CdSeTe interfaces for photovoltaics. IEEE J. Photovolt..

[12] Ren S., Li H., Lei C., Li C., Yin X., Wu L., Li W., Zhang J., Wang W., Feng L. (2019). Interface modification to enhance electron extraction by deposition of a ZnMgO buffer on SnO_2_-coated FTO in CdTe solar cells. Sol. Energy.

[13] Awni R.A., Li D.-B., Song Z., Bista S.S., Razooqi M.A., Grice C.R., Chen L., Liyanage G.K., Li C., Phillips A.B. (2019). Influences of buffer material and fabrication atmosphere on the electrical properties of CdTe solar cells. Prog. Photovolt. Res. Appl..

[14] Kephart J.M., McCamy J.W., Ma Z., Ganjoo A., Alamgir F.M., Sampath W.S. (2016). Band alignment of front contact layers for high-efficiency CdTe solar cells. Sol. Energy Mater. Sol. Cells.

[15] Li D.-B., Song Z., Awni R.A., Bista S.S., Shrestha N., Grice C.R., Chen L., Liyanage G.K., Razooqi M.A., Phillips A.B. (2019). Eliminating s-kink to maximize the performance of MgZnO/CdTe solar cells. ACS Appl. Energy Mater..

[16] Ohtomo A., Kawasaki M., Koida T., Masubuchi K., Koinuma H. (1998). Mg*_x_*Zn_1−*x*_O as a II–VI widegap semiconductor alloy. Appl. Phys. Lett..

[17] Minemoto T., Negami T., Nishiwaki S., Takakura H., Hamakawa Y. (2000). Preparation of Zn_1−*x*_Mg*_x_*O films by radio frequency magnetron sputtering. Thin Solid Films.

[18] Hsu H.-C., Wu C.-Y., Cheng H.-M., Hsieh W.-F. (2006). Band gap engineering and stimulated emission of ZnMgO nanowires. Appl. Phys. Lett..

[19] Ferlito E.P., Ricciari R., Padalino M., Grasso S., Battaglia A., Sciuto M., Mello D., Tapfer L., Gerardi C. (2014). Determination of Mg concentration and distribution in Mg*_x_*Zn_1−*x*_O films for photonic devices application. Surf. Interface Anal..

[20] Amarasinghe M., Colegrove E., Moseley J., Moutinho H., Albin D., Duenow J., Jensen S., Kephart J., Sampath W. (2018). Obtaining large columnar CdTe grains and long lifetime on nanocrystalline CdSe, MgZnO, or CdS layers. Adv. Energy Mater..

[21] Rao G.V., Säuberlich F., Klein A. (2005). Influence of Mg content on the band alignment at CdS/(Zn,Mg)O interfaces. Appl. Phys. Lett..

[22] Minemoto T., Hashimoto Y., Shams-Kolahi W., Satoh T., Negami T., Takakura H., Hamakawa Y. (2014). Control of conduction band offset in wide-gap Cu(In,Ga)Se_2_ solar cells. Sol. Energy Mater. Sol. Cells.

[23] Ablekim T., Colegrove E., Metzger W.K. (2018). Interface engineering for 25% CdTe solar cells. ACS Appl. Energy Mater..

[24] Messier R., Giri A.P., Roy R.A. (1984). Revised structure zone model for thin film physical structure. J. Vac. Sci. Technol. A.

[25] Mayes E.L.H., Murdoch B.J., Bilek M.M.M., McKenzie D.R., McCulloch D.G., Partridge J.G. (2015). Co-deposition of band-gap tuned Zn_1−*x*_Mg*_x_*O using high impulse power and dc-magnetron sputtering. J. Phys. D Appl. Phys..

[26] Coppa B.J., Davis R.F., Nemanich R.J. (2003). Gold Schottky contacts on oxygen plasma-treated, *n*-type ZnO(0001). Appl. Phys. Lett..

[27] Kim S., Lee C.-S., Kim S., Chalapathy R.B.V., Al-Ammar E.A., Ahn B.T. (2015). Understanding the light soaking effect of ZnMgO buffer in CIGS solar cells. Phys. Chem. Chem. Phys. (Inc. Faraday Trans.).

[28] Ren S., Wang H., Li Y., Li H., He R., Wu L., Li W., Zhang J., Wang W., Feng L. (2018). Rapid thermal annealing on ZnMgO window layer for improved performance of CdTe solar cells. Sol. Energy Mater. Sol. Cells.

[29] Wang A., Tang T., Ren S., Zhang J., Wu L., Li W., Wang W., Feng L. (2019). Characterization of co-sputtered Mg*_x_*Zn_1−*x*_O thin films and their application in CdTe solar cells. Mater. Sci. Semicond. Process..

[30] Li H., Zhang Y., Pan X., Wang T., Xie E. (2009). The effects of thermal annealing on properties of Mg*_x_*Zn_1−*x*_O films by sputtering. J. Alloys Compd..

[31] Kim J.W., Kang H.S., Kim J.H., Lee S.Y. (2006). Variation of structural, electrical, and optical properties of Zn_1−*x*_Mg*_x_*O thin films. J. Appl. Phys..

[32] Artegiani E., Leoncini M., Barbato M., Meneghini M., Meneghesso G., Cavallini M., Romeo A. (2019). Analysis of magnesium zinc oxide layers for high efficiency CdTe devices. Thin Solid Films.

[33] Baines T., Durose K., Major J.D. (2018). Co-sputtered Mg*_x_*Zn_(1−x)_O window layers for CdTe_(1−*x*)_Se*_x_* solar cells. Proceedings of the 7th World Conference on Photovoltaic Energy Conversion (WCPEC) (A Joint Conference of 2018 IEEE 45th PVSC, 28th PVSEC & 34th EU PVSEC).

[34] Bittau F., Potamialis C., Togay M., Abbas A., Isherwood P.J.M., Bowers J.W., Walls J.M. (2018). Analysis and optimisation of the glass/TCO/MZO stack for thin film CdTe solar cells. Sol. Energy Mater. Sol. Cells.

[35] Bittau F., Artegiani E., Abbas A., Menossi D., Romeo A., Bowers J.W., Walls J.M. (2017). Magnesium-doped zinc oxide as a high resistance transparent layer for thin film CdS/CdTe solar cells. Proceedings of the 2017 IEEE 44th Photovoltaic Specialists Conference (PVSC).

[36] Ke Y., Berry J., Parilla P., Zakutayev A., O’Hayre R., Ginley D. (2012). The origin of electrical property deterioration with increasing Mg concentration in ZnMgO:Ga. Thin Solid Films.

[37] Zhang X., Ma H., Wang Q., Ma J., Zong F., Xiao H., Ji F., Hou S. (2005). Structural and optical properties of Mg*_x_*Zn_1−*x*_O thin films deposited by magnetron sputtering. Phys. B Condens. Matter.

[38] Guan W.L., Lian J., Yu Y.X., Sun Z.Z., Zhao M.L., Wang X., Zhang W.F. (2014). Optical properties of Mg*_x_*Zn_1−*x*_O thin films deposited on silicon and sapphire substrate by rf magnetron sputtering. Optik.

[39] Koster R.S., Fang C.M., Dijkstra M., van Blaaderen A., van Huis M.A. (2015). Stabilization of rock salt ZnO nanocrystals by low-energy surfaces and Mg additions: A first-principles study. J. Phys. Chem. C.

[40] Kutlu-Narin E., Narin P., Yildiz A., Lisesivdin S.B. (2021). Effect of magnesium content and growth temperature on structural and optical properties of USCVD-grown MgZnO films. Appl. Phys. A.

[41] Fan X.F., Sun H.D., Shen Z.X., Kuo J.-L., Lu Y.M. (2008). A first-principle analysis on the phase stabilities, chemical bonds and band gaps of wurtzite structure A*_x_*Zn_1−*x*_O alloys (A = Ca, Cd, Mg). J. Phys. Condens. Matter.

[42] Choopun S., Vispute R.D., Yang W., Sharma R.P., Venkatesan T. (2002). Realization of band gap above 5.0 eV in metastable cubic-phase Mg*_x_*Zn_1−*x*_O alloy films. Appl. Phys. Lett..

[43] Maznichenko I.V., Ernst A., Bouhassoune M., Henk J., Däne M., Lüders M., Bruno P., Hergert W., Mertig I., Szotek Z. (2009). Structural phase transitions and fundamental band gaps of Mg*_x_*Zn_1−*x*_O alloys from first principles. Phys. Rev. B.

[44] Kang J.H., Park Y.R., Kim K.J. (2000). Spectroscopic ellipsometry study of Zn_1−*x*_Mg*_x_*O thin films deposited on Al_2_O_3_(0001). Solid State Commun..

[45] Cuong H.B., Le N.M., Jeong S.-H., Lee B.-T. (2017). Tailoring of composition, band-gap, and structural phase in ZnMgO films by simply controlling growth temperature and oxygen partial pressure during sputter deposition. J. Alloys Compd..

[46] Fujiwara H., Koh J., Rovira P.I., Collins R.W. (2000). Assessment of effective-medium theories in the analysis of nucleation and microscopic surface roughness evolution for semiconductor thin films. Phys. Rev. B.

[47] Collins R.W., Vedam K., Trigg G.L. (1995). Optical properties of solids. Encyclopedia of Applied Physics.

[48] Collins R.W., Ferlauto A.S., Tompkins H.G., Irene E.A. (2005). Optical physics of materials. Handbook of Ellipsometry.

[49] Koirala P., Li J., Podraza N.J., Collins R.W., Fujiwara H., Collins R.W. (2018). Real time and mapping spectroscopic ellipsometry for CdTe photovoltaics. Spectroscopic Ellipsometry for Photovoltaics; Fundamental Principles and Solar Cell Characterization.

[50] Schmidt-Grund R., Schubert M., Rheinländer B., Fritsch D., Schmidt H., Kaidashev E.M., Lorenz M., Herzinger C.M., Grundmann M. (2004). UV–VUV spectroscopic ellipsometry of ternary Mg*_x_*Zn_1−*x*_O (0 ≤ *x* ≤ 0.53) thin films. Thin Solid Films.

[51] Koirala P., Li J., Yoon H.P., Aryal P., Marsillac S., Rockett A.A., Podraza N.J., Collins R.W. (2016). Through-the-glass spectroscopic ellipsometry for analysis of CdTe thin-film solar cells in the superstrate configuration. Prog. Photovolt. Res. Appl..

[52] Niklasson G.A., Granqvist C.G., Hunderi O. (1981). Effective medium models for the optical properties of inhomogeneous materials. Appl. Opt..

[53] Koh J., Lu Y., Wronski C.R., Kuang Y., Collins R.W., Tsong T.T., Strausser Y.E. (1996). Correlation of real time spectroellipsometry and atomic force microscopy measurements of surface roughness on amorphous semiconductor thin films. Appl. Phys. Lett..

[54] Dahal L.R., Sainju D., Podraza N.J., Marsillac S., Collins R.W. (2011). Real time spectroscopic ellipsometry of Ag/ZnO and Al/ZnO interfaces for back-reflectors in thin film Si:H photovoltaics. Thin Solid Films.

[55] Collins R.W., Koirala P., Podraza N.J., Podraza N.J., Collins R.W. (2021). Near-infrared to ultraviolet optical response and the absorption onset: Parametric representations. The World Scientific Reference of Amorphous Materials; Structure, Properties, and Applications of Tetrahedrally Bonded Thin-Film Amorphous Semiconductors.

[56] Oueslati M., Zouaghi M., Pistol M.E., Samuelson L., Grimmeiss H.G., Balkanski M. (1985). Photoluminescence study of localization effects induced by the fluctuating random alloy potential in indirect band-gap GaAs_1−*x*_P*_x_*. Phys. Rev. B.

[57] Huso J., Che H., Thapa D., Canul A., McCluskey M.D., Bergman L. (2015). Phonon dynamics and Urbach energy studies of MgZnO alloys. J. Appl. Phys..

[58] Chen J., Li J., Sainju D., Wells K.D., Podraza N.J., Collins R.W. (2006). Multilayer analysis of the CdTe solar cell structure by spectroscopic ellipsometry. Proceedings of the 4th World Conference on Photovoltaic Energy Conversion (WCPEC).

[59] Koirala P., Paudel N., Chen J., Attygalle D., Yan Y., Collins R.W. (2012). Real time and post-deposition optical analysis of interfaces in CdTe solar cells. Proceedings of the 2012 IEEE 38th Photovoltaic Specialists Conference.

[60] Koirala P. (2015). Multichannel Spectroscopic Ellipsometry for CdTe Photovoltaics: From Materials and Interfaces to Solar Cells. Ph.D. Thesis.

